# Impact of SARS-CoV-2 Epidemic on Antimicrobial Resistance: A Literature Review

**DOI:** 10.3390/v13112110

**Published:** 2021-10-20

**Authors:** Francesco Vladimiro Segala, Davide Fiore Bavaro, Francesco Di Gennaro, Federica Salvati, Claudia Marotta, Annalisa Saracino, Rita Murri, Massimo Fantoni

**Affiliations:** 1 Clinic of Infectious Diseases, Catholic University of the Sacred Heart, 00168 Rome, Italy; fedes-95@hotmail.it (F.S.); rita.murri@unicatt.it (R.M.); massimo.fantoni@policlinicogemelli.it (M.F.); 2 Clinic of Infectious Diseases, University of Bari, 70121 Bari, Italy; davidebavaro@gmail.com (D.F.B.); cicciodigennaro@yahoo.it (F.D.G.); annalisa.saracino@uniba.it (A.S.); 3 General Directorate of Health Prevention, Ministry of Health, 00144 Rome, Italy; marotta.claudia@gmail.com; 4 Dipartimento di Scienze di Laboratorio e Infettivologiche, Fondazione Policlinico Universitario A. Gemelli IRCCS, 00168 Rome, Italy

**Keywords:** COVID-19, antimicrobial resistance, infection control, *Candida auris*, carbapenem-resistant *Acinetobacter baumannii*, carbapenem-resistant Enterobacteriaceae, methicillin-resistant *Staphylococcus aureus*

## Abstract

Antimicrobial resistance is an urgent threat to public health and global development; in this scenario, the SARS-CoV2 pandemic has caused a major disruption of healthcare systems and practices. A narrative review was conducted on articles focusing on the impact of COVID-19 on multidrug-resistant gram-negative, gram-positive bacteria, and fungi. We found that, worldwide, multiple studies reported an unexpected high incidence of infections due to methicillin-resistant *S. aureus*, carbapenem-resistant *A. baumannii*, carbapenem-resistant Enterobacteriaceae, and *C. auris* among COVID-19 patients admitted to the intensive care unit. In this setting, inappropriate antimicrobial exposure, environmental contamination, and discontinuation of infection control measures may have driven selection and diffusion of drug-resistant pathogens.

## 1. Introduction. Overview on Antimicrobial Resistance Trends Prior to COVID-19 Pandemic

Antimicrobial resistance (AMR), in the last two decades, has grown at such an extension that it has been recognized as one of the most urgent threats facing global health and economic development [1,2]. In settings with a high rate of antimicrobial prescription such as medicine wards and intensive-care units, infections by difficult-to-treat bacteria are increasingly associated by elevated mortality [3] and high hospital costs [4,5]. The European Centre for Disease Prevention and Control (ECDC) reported more than 670,000 infections and 33,000 deaths due to resistant bacteria in the course of 2019 [6], while, only in the United States, resistant bacteria and fungi were responsible for at least 3 million new infections and 35,900 deaths each year [7].

In the latest report from the ECDC, the most common resistant bacteria was *E. coli*, followed by *S. aureus*, *K. pneumoniae*, *E. faecalis,* and *P. aeruginosa* [6]. *E. coli* was also the most reported resistant pathogen causing bloodstream infection (BSI) worldwide, with 40% of isolates resistant to third-generation cephalosporins (ESBL-*E. coli*) [8,9]. Resistance rates were similar for urinary tract infections, with few specimens showing resistance to carbapenems. On the other hand, mean resistance against carbapenems was remarkably high for *K. pneumoniae* (7.9%), *P. aeruginosa* (16.5%), and *Acinetobacter* spp. (32%) [6]. However, rates of carbapenem resistance varied greatly in different geographical areas, reaching levels as high as 58% for *K. pneumoniae* and 92% for *A. baumannii* in regions like Southeastern Europe, both showing an epidemiological trend towards increase [7,10]. In particular, the emergence and spread of carbapenem-resistant strains of *A. baumannii* is concerning, since carbapenems were used as last-resort antibiotics for critically ill patients until recently. In addition, once it has become endemic, *A. baumannii* is also a challenging pathogen to eradicate from healthcare settings [11] because of its capacity to survive on moist fomites like environmental surfaces or contaminated devices (such as ventilators) [12,13]. Nevertheless, despite its ability to accumulate mechanisms of resistance and to survive in hospital environments, before the COVID-19 pandemic, *A. baumannii* was responsible for only 1.7% of infections due to resistant pathogens reported by the ECDC [6].

Among gram-positive bacteria, methicillin-resistant *S. aureus* (MRSA) is the most common bacteria reported by surveillance systems in Europe, and the most common pathogen causing skin and soft tissue infection presenting to US hospital emergency departments [14]. Incidence of MRSA infection varies consistently, reaching its highest rates in Latin America, Japan, and Southeastern Europe, but, globally, resistance to penicillins accounts for 24.9% of *S. aureus* isolates [15,16]. Along with ESBL-*E. coli*, MRSA is included as an AMR indicator for the Sustainable Development Goals developed by the World Health Organization [17], and incidence of MRSA infections appears to show a decreasing trend in recent decades [6,7] but, when compared for income level, low- and middle-income countries showed a markedly higher rate of antimicrobial resistance [8]. Unlike MRSA, however, infections due to other resistant gram-positive bacteria, like vancomycin-resistant enterococci (i.e., vancomycin-resistant *E. faecium*), are demonstrating an alarming increase in some European countries (i.e., Portugal, Spain, Italy, and Greece) and in several regions of South America [18]. 

Among fungi, an emerging pathogen causing concern worldwide is drug resistant *Candida* spp. More specifically, *C. auris* has recently been recognized as an “urgent threat” by the CDC [7] as it has been reported in more than 32 countries since it was first described in Japan one decade ago [19]. In fact, in addition to its fast spreading and its ability to develop resistance to all known antimycotic drugs [20], infection with *C. auris* is associated with high mortality rates, rapid in-hospital spread, and challenging laboratory identification [21]. Patients at particular risk for infection due to *C. auris* are those who underwent recent abdominal surgery or those with a history of wide-spectrum antimicrobial exposure.

In this complex scenario, the pandemic caused by severe acute respiratory syndrome coronavirus 2 (SARS-CoV2) has disrupted healthcare systems and practices all over the world. Its impact on antimicrobial resistance is still largely unknown but, as a part of the response, there is evidence that antimicrobial prescription habits have been profoundly affected [22]. The aim of this review was to investigate whether, in the available literature, it is possible to identify new trends in antimicrobial resistance that may be a consequence of the COVID-19 pandemic.

## 2. Materials and Methods

We conducted a search on PubMed, Scopus, Google Scholar, EMBASE, Cochrane Library, and WHO websites (http://www.who.int, accessed on 7 September 2021) from March 2020 to August 2021 in order to identify articles discussing the role of the COVID-19 pandemic on antimicrobial resistance. We structured the research on methicillin-resistant *S. aureus*, drug-resistant *Enterococcus* spp., extended-spectrum beta lactamase producing Enterobacteriaceae, carbapenem-resistant Enterobacteriaceae, carbapenem-resistant *A. baumannii*, carbapenem-resistant *P. aeruginosa*, azole-resistant *Aspergillus* spp., and drug-resistant *Candida* spp. The pathogens were selected in accordance with their identification as “threats” by the CDC [7], to their potential to promote nosocomial clusters, and, in case of *Aspergillus* spp., because of its recognized association with COVID-19 infections.

## 3. Results

Since the early days of the SARS-CoV-2 outbreak, questions have been raised regarding the risk of bacterial co-infections and bacterial super-infections in the course of COVID-19. Importantly, co-infections are usually defined as all other diseases caused by a pathogen different from SARS-CoV-2 coexisting at the time of COVID-19 diagnosis, while secondary infections are defined as all infective events that were absent at presentation, but which occurred in the course of the disease [23]. Below, we discuss the available evidence about infections due to resistant gram-positive, gram-negative bacteria, and fungi among hospitalized COVID-19 patients. The main results are summarized in Table 1.

### 3.1. Multidrug Resistant Gram-Positive Bacteria

#### 3.1.1. Methicillin-Resistant Staphylococcus Aureus

Gram-positive infections were particularly feared in the course of severe COVID-19 cases requiring ICU admission because of the experience derived from severe staphylococcal pneumonia in the course of Influenza [24].

However, data from a large meta-analysis demonstrated that bacterial co-infection occurred in less than 10% of all subjects hospitalized with COVID-19 [25], with a higher incidence in those with a severe disease. In this setting, common gram-positive pathogens, including *S. pneumoniae,* were particularly frequent causes of lung infections, as observed in the general population. Moreover, as anticipated, multiple case series also documented a significant incidence of methicillin-sensitive (MSSA) and methicillin-resistant *Staphylococcus aureus* (MRSA) infections in bronchoalveolar lavage of subjects admitted to the ICU [26], justifying the use of anti-MRSA drugs in an empirical approach of severe pneumonia in the course of COVID-19 with clinical or biochemical markers suggestive of bacterial secondary infection. Therefore, up to 86.4% of all COVID-19 patients admitted to the ICU received a wide spectrum antibiotic therapy [22] in order to provide an adequate coverage against possible bacterial lung coinfections. This approach could virtually resolve the vast majority of coinfections, but produced a dramatic selective pressure and exposed a large number of patients to unnecessary antibiotics. In any case, it should be considered that the presence of MRSA pulmonary coinfections reflects the usual epidemiology of different countries and was less influenced by the COVID-19 pandemic itself. For instance, in a large case series conducted in the US, a low prevalence of MRSA nasal colonization was found, in accordance with the decreasing local incidence [27] even during an important pandemic wave. In contrast, data from a large meta-analysis showed a significant influence of MRSA co-infection on COVID-19 mortality, especially in the case of co-infections, causing a significant number of ICU admissions [28].

Moreover, excluding coinfections, emerging data confirmed the importance of MRSA as a potential agent of secondary infections, including ventilator-associated pneumonia (VAP) in subjects hospitalized in ICU for SARS-CoV-2 respiratory failure [29] and severe bloodstream infections [30], significantly increasing the overall risk of mortality. In fact, a dramatically high 14-day and 30-day mortality (54.8% and 66.7%, respectively) was documented in a recently published case series of *S. aureus* bloodstream infections [31]. Interestingly, in this series, 69% of bloodstream infections were from an unknown source. Conversely, among the remaining cases, the principal site of origin was the lung (19%), followed by endovascular (7.2%), bone and joints (2.4%), and skin/soft tissue (2.4%).

#### 3.1.2. Multidrug-Resistant *Enterococcus* spp.

The wide use of antibiotics in subjects admitted for COVID-19 [32], specifically cephalosporins and glicopeptides, along with producing selective pressure on the hospital environment [33], is a recognized determinant of both strain selection and incidence of infections due to ampicillin-resistant, vancomycin-resistant *Enterococcus* spp. [34,35]. Additionally, being major microbiome disruptors and recognized risk factors for bacterial translocation [36], antibiotics may expose ICU patients to an additional risk of secondary BSIs from gut pathogens. In fact, in a study conducted by Tang et al., the abundance of *Enterococcus* spp. was reported to increase in the gut microbiota of adult COVID-19 patients with poor prognosis [37].

In COVID-19 settings, selective pressure could be evident both at the patient and environmental level, as in the case of a VRE cluster described in a German ICU that was confirmed by genome multilocus sequence typing analysis [38]. Additionally, in a retrospective cohort of 89 ICU patients, Bonazzetti et al. reported an unexpectedly high incidence of primary and catheter-associated bloodstream infections caused by *E. faecalis* and *E. faecium* [39] and, similarly, the incidence of bloodstream infections caused by gram-positive bacteria, including multidrug resistant *Enterococci*, significantly increased mortality and length of ICU stay in a retrospective cohort of 140 patients in Spain [40]. In all studies, a disruption in infection control measures was identified as the main causative agent of the outbreak.

### 3.2. Gram Negative Bacteria

Multidrug-resistant gram-negative organisms (MDRGN) colonization or superinfections may be a possible complication of ICU stays. In the months of the early pandemic, controlling COVID-19 transmission and managing patients with COVID-19 effectively was emphasized, causing a shift of infection control and antimicrobial stewardship focus of care that, in turn, may have led to an increased incidence of MDRGNs colonization and infections [41]. A particular concern was caused by the increasing incidence of carbapenem-resistant Enterobacteriaceae (CRE) [42] and non-fermenting MDRGNs like carbapenem-resistant *Acinetobacter baumannii* (CRAB) [43] and *Pseudomonas aeruginosa* [44].

#### 3.2.1. Carbapenem-Resistant Enterobacteriaceae

According to a recent review on CRE infections during COVID-19, exploring in particular the secondary infections caused by CR-*Klebsiella pneumoniae* (CR-Kp), the prevalence of coinfection in COVID-19 patients varied greatly, ranging from 0.35% to 53%. Importantly, the most frequently isolated resistance gene was KPC, followed by OXA-48 and NDM, being pneumonia and BSI the main source of infection. In contrast, urinary tract, intra-abdominal, and skin and soft tissue infections were very rare, probably because the main drivers of dissemination of CR-Kp were identified to be the use of mechanical ventilation and central catheters [45].

To this regard, Yang et al. [46] observed a higher rate of CRE colonization in patients where positioning (prone position) was used in the treatment process, if compared with patients who were not prone-positioned (67% vs. 37%). Despite the lack of a clear explanation for this result, it is possible that a combination of wide antibiotic prescription, higher severity of prone-positioned patients, and employment of additional staff who often had no experience of working in the ICU [47] caused this phenomenon. 

It should be noticed that CR-Kp colonization and infections were associated with a high rate of mortality in multiple reports [45,46,48], representing a significant clinical challenge in terms of both infection control and clinical practice, despite the availability of new and effective antimicrobials for CRE. In any case, an increased use of antimicrobial agents active against these pathogens was reported in many countries during the current pandemic, inducing further issues in terms of antimicrobial stewardship. Interestingly, the wide spread of KPC-Kp infections and the overuse of new beta-lactams/beta-lactamase inhibitors has already shown to induce further selection and spreading of multidrug-resistant associated genes in ICUs, like metallo-beta-lactamase, including blaNDM and blaVIM [49,50], or selection of hypervirulent KPC-Kp resistant to ceftazidime/avibactam [51].

Overall, these data indicate the need for high attention to infections caused by CR-Kp, the predominant nosocomial pathogen among CRE, specifically in patients with critical COVID-19 in whom the immune system undergoes a complex pattern of immune dysregulation [52].

#### 3.2.2. Carbapenem-Resistant Non-Fermenting Gram-Negative Bacteria

Along with CRE, another important nosocomial threat is represented by CRAB secondary infections. To date, multiple outbreaks of CRAB have been reported in COVID-19-dedicated hospitals [43,53,54,55,56,57]; in addition, a recent report from Italy demonstrated that the overall incidence of CRAB infections increased from 5.1 per 10,000 ICU-patient-days in January–April 2019 to 26.4 per 10,000 ICU-patient-days in January–April 2020, suggesting a worsening of CRAB spreading in ICUs strictly correlated with the occurrence of the COVID-19 pandemic [58]. Notably, CRAB infections and colonization represent an important risk factor for morbidity and mortality due to the extensive antimicrobial resistance spectrum of these bacteria and are a renowned complication of prolonged ICU stays. However, emerging data showed an independent effect of CRAB on risk of mortality in COVID-19 patients if compared with subjects hospitalized in ICUs for other medical conditions [54]. Importantly, in the setting of COVID-19, an additional risk factor for developing an MDR infection or for mortality after an infection was the corticosteroid therapy and immunomodulatory drugs, such as tocilizumab, baricitinib, etc. In fact, these agents are associated with a well-known immunosuppressive effect [59] that could facilitate secondary infections [60] or directly influence the response to bacterial dissemination [54].

Lastly, CRAB infection-related mortality is highly influenced by the low response to antimicrobials, particularly in the setting of critically ill patients. In fact, the backbone for treatment of CRAB infections is still represented by colistin, an antibiotic burdened by high toxicity and unpredictable pharmacokinetics/pharmacodynamics properties, and clinical data of new therapeutic options, such as cefiderocol, are still urgently needed [61,62]. 

Excluding CRE and CRAB, severe infections by other non-fermenting gram-negative bacteria have been reported in patients with COVID-19. 

Among these, *Stenotrophomonas maltophilia* should be considered a pathogen of interest due to its limited treatment options [63] and the fact that it typically infects patients with ongoing prolonged mechanical ventilation and extensive exposure to antibiotics [64]. Additionally, reports of *S. maltophilia* were particularly frequent in highly immunocompromised patients [65] or subjects with particularly complicated clinical courses with multiple subsequent secondary infections and severe lung failure [66]. Yet, *S. maltophilia* represents more often an opportunistic pathogen, rather than a “simple” causative agent of nosocomial infection, suggesting the need of a high index of suspicion. Nevertheless, the description of *S. maltophila* infections and is still anectodical, and the exact incidence is unknown and does not appear to be significantly higher in COVID-19 patients.

On the other side, several studies reported the occurrence of carbapenem-resistant *P. aeruginosa* (CRPA) in critically ill COVID-19 patients [48,67]. However, incidence of CRPA varied among different studies, and in-hospital outbreaks have not been reported yet. This is probably due to intrinsic characteristics of *P. aeruginosa* in terms of virulence, adaptability, and spreading features. Biofilm-forming properties of *P. aeruginosa* may be the cause of problematic chronic lung infections, particularly in COVID-19 patients who underwent mechanical ventilation [68]. In any case, differently from CRE and CRAB, current literature did not show a significant spread of CRPA in course of COVID-19, although it could still represent a major threat for some patients.

### 3.3. Fungi

Critically ill COVID-19 presents several major risk factors for invasive fungal infections, such as mechanical ventilation, prolonged ICU stays, older age, protracted corticosteroid therapy, and extensive antimicrobial exposure [46,69]. Several fungal pathogens have been reported to challenge the clinical management of COVID-19 patients such as mucoromycetes [70] but, in this review, we focused on available data about antimycotic resistance in COVID-19-associated pulmonary aspergillosis (CAPA) and COVID-19-associated candidemia (CAC).

#### 3.3.1. Triazole-Resistant *Aspergillus* spp. in COVID-19-Associated Pulmonary Aspergillosis

COVID-19-associated pulmonary aspergillosis (CAPA) is a recently described disease entity affecting patients admitted in intensive care units, with incidence rates ranging from 12.3% to 33.3% [71,72,73,74,75] and a mortality rate approaching 50%. Along with the severity of the clinical presentation, a major challenge is represented by its recognition, since clinical and radiological features are similar to COVID-19-related respiratory distress syndrome [76].

Recently, two fatal cases of superinfections by multi-azole resistant *A. fumigatus* have been reported in severe COVID-19 patients in the Netherlands [77] and Ireland [78]. In these reports, both *A. fumigatus* isolates harbored the TR34/L98H mutation, which is the most prevalent environmental exposure mechanism of resistance acquisition, commonly resulting in panazole-resistance [79], while they were still susceptible to amphotericin B. However, effective antifungal treatment was initiated only when antimicrobial susceptibility test (AST) became available. Besides, the same mutation has been identified in France [80] in a COVID-19 patient who presented negative screening for fungal infections at the time of admission to the ICU. Like in the previous reports, the patient died, but the clinical deterioration was so rapid that antifungal treatment was not yet started. In all cases, prior to CAPA diagnosis, patients were exposed to at least one week of broad-spectrum antibiotic therapy.

Due to the scarcity of data, it is unknown whether these reports are an expression of an increased incidence of antimicrobial resistance or a simple expression of local ecology. Evaluation of resistance epidemiology is also hampered by the fact that resistance testing is not widespread. Nevertheless, the finding among COVID-19 patients of three geographically unrelated reports of *A. fumigatus* harboring the same mutation conferring panazole resistance is worth consideration.

#### 3.3.2. Multidrug Resistant *Candida* spp. in COVID-19-Associated Candidemia

Since January 2020, an increasing number of studies have reported a higher incidence of candidemia among critically ill COVID-19 patients, with reported mortality rates as high as 46% [81]. Moreover, in some ICU settings, the emergence of COVID-19-associated candidemia (CAC) was complicated by the presence of drug-resistant Candida isolates, such as *C. auris*, and multi-drug resistant strains of *C. albicans*. In fact, risk factors for resistant Candida infections are common among critically ill COVID-19 patients and include long ICU stay, respiratory disease, presence of a central iv line, indwelling urinary catheters, and prolonged exposure to antifungal drugs [82].

During the ongoing COVID-19 pandemic, the large number of COVID-19 patients requiring critical care and the prolonged use of personal protective equipment (PPE) by the healthcare personnel may have inadvertently mediated a silent dissemination of this fungal pathogen [83,84]. For instance, in an epidemiological investigation conducted in Brazil, Nobrega de Almeida et al. found that axillary monitoring thermometers may facilitate the dissemination of *C. auris* in hospital settings [85].

In a study conducted in New Delhi, *C. auris* was the main causative agent of candidemia among critically ill COVID-19 patients admitted to an intensive care unit (10/15, 67%) [86] and, among those, the fatality rate was 60%. Of note, in this study, four of the six patients who died experienced persistent fungemia despite echinocandin treatment. Antifungal susceptibility testing data for *C. auris* isolates showed that resistance rates to fluconazole, voriconazole, amphotericin B and 5-flucytosinewere, respectively, 100%, 30%, 40%, and 60%. Overall, three *C. auris* isolates were multiazole (fluconazole + voriconazole) resistant, whereas seven were multidrug resistant, including three that were resistant to three classes of antifungals (azoles + amphotericin B + 5-flucytosine) and four isolates resistant to classes classes of drugs (azoles + 5-flucytosine and azoles + amphotericin B). All isolates were susceptible to echinocandins. In this report, authors observed that incorrect and extended use of personal protective equipment may have played a role in self-contamination and transmission of *C. auris* among COVID-19 patients. 

In another case series, Villanueva-Lozano et al. documented an outbreak of *C. auris* in Mexico, which started in a non-COVID-19 patient, and later spread to 12 patients admitted into a COVID-19 ICU [87]. *C. auris* was isolated from the blood samples in six patients, from urine in eight, and from both sites in two. In this study, among patients with candidemia, mortality was 83.3%. Antifungal susceptibility testing showed that all the isolates were resistant to AMB just one isolate was resistant to ANF, one to CAS and eight isolates were resistant to FLU. Eight isolates were resistant to at least two major classes of antifungals.

Furthermore, a retrospective, multicenter study conducted in Iran reported the case of six patients affected by bloodstream infection due to resistant *C. albicans* [81]. Even though *C. albicans* is known as the species with the lowest rate of drug resistance, half of the isolates reported in this study showed resistance to both azoles and echinocandins and were treated first with fluconazole and then by caspofungin, which ultimately led to therapeutic failure. Thus, this study was the first to identify multidrug-resistant *C. albicans* among patients with CAC.

## 4. Discussion. Disruption of Infection Control Measures and Antimicrobial Stewardship in COVID ICUs

Though the available evidence is low quality (deriving mainly from case reports, case series and monocentric observational studies), making it difficult to assess the precise extent to which COVID-19 pandemic impacted antimicrobial resistance, it is reasonable to believe that the widespread disruption of healthcare services led to a significant shift of AMR patterns worldwide. In particular, the increased patient exposure to antimicrobials, along with an unprecedented burden on healthcare workers, limited laboratory capacity, and a diffuse loss of adherence to routine infection control practices (including proper screening and isolation of patients colonized by MDROs) are likely to have promoted the selection and diffusion of resistant pathogens [88]. Concerning antimicrobial prescription, large meta-analyses have identified an important gap between the incidence of co- and secondary infections (respectively 3.5% and 14.5%) [23], and the proportion of COVID-19 patients who were exposed to antimicrobials [22], that peaked to 86.4% among critically ill patients. To our knowledge, this is the first review discussing the role of COVID-19 pandemic on the incidence of infection due to specific resistant bacterial pathogens. At the same time, it is the first review that explores the role of COVID-19 on the epidemiology of drug-resistant fungi.

In this narrative review evaluating the impact of the ongoing pandemic on antimicrobial resistance, we found that the vast majority of the reports came from intensive care units. This does not mean that COVID-19 did not affect AMR patterns in general wards, but it may indicate that, at the time of writing, the impact of the pandemic on antimicrobial resistance was somehow more pronounced inside the ICU. On one hand, this assumption is supported by the evidence that antimicrobial prescribing was higher in the ICU than in general wards as, in the early phase of the pandemic, empiric broad-spectrum antibiotics were included as part of the recommended therapy for all critically ill COVID-19 patients admitted to the ICU [89,90]. More specifically, in October 2020, the European Society of Clinical Microbiology and Infectious Diseases (ESCMID) published dedicated guidelines on proper use of antibiotics in COVID-19 that endorsed the recommendation of the 2020 Surviving Sepsis Campaign guideline on COVID-19 to treat critically ill patients admitted to the ICU for COVID-19 with empiric antibiotic therapy while awaiting test results [91]. On a clinical standpoint, SARS-CoV-2 infection and bacterial pneumonia show a similar presentation and sometimes overlapping radiological appearance, which makes it is difficult to determine what patient should, or should not, receive antibiotics [92]. However, even when broad-spectrum antibiotics are given empirically to all mechanically ventilated patients, prescriptions should be assessed for de-escalation on a daily basis, in consideration of patient’s clinical status and laboratory results, and there is reason to believe that adherence to this practice can be consistently improved [93]. A well-known consequence of antibiotic overprescription is *C. difficile* infection, which may complicate itself the clinical course of COVID-19, particularly for patients with co-morbidities and previous healthcare exposure [94,95].

On the other hand, however, reports of clusters of infections due to the same resistant pathogen raise the concern of a decline in adherence to infection control and prevention measures. In our review, we found that special attention should be regarded to *A. baumannii.* [38], *C. auris* [83], and MDR gram-negative Enterobacteriaceae [45]. Circumstances like overcrowding, multiple staff in contact with each patient (as for the application of prone-positioning), and low-compliance with contact isolation practices (like glove hygiene and gown changes) may promote the diffusion of the resistant pathogens among the same ICU [96]. Another aspect that deserves special consideration is the importance of environmental contamination. In the setting of a COVID-19 ICU, fomite transmission, which is challenging both to recognize and to eradicate [97], was identified to be responsible for nosocomial transmission of KPC-Kp in Germany (ventilators), and in Brazil C. auris (axillary thermometers) [85]—and it is a well-known source of nosocomial outbreaks of MRSA [15] and CRAB [12]. In New Jersey, during a peak of COVID-19 cases in April 2020, Perez et al. reported several unintentional changes in standard infection prevention and control (IPC) practices, which included a temporary discontinuation of appropriate PPE use, environmental cleaning and prolonged use of ventilator circuits and suctioning catheters [98]. Furthermore, in Italy, in a perspective case-control study conducted by De Pascale et al. on mechanically ventilated patients, the proportion of ventilator associated pneumonia due to methicillin-resistant vs. methicillin susceptible S. aureus was significantly higher for COVID-19 patients (65.0% vs. 27.5%; *p* < 0.01) vs. non-COVID-19 patients, suggesting a higher rate of MRSA colonization/environmental contamination in COVID-19 ICUs [29].

The present review has several obvious limitations. First, being a narrative review, it lacked a pre-defined protocol, and thus a selection bias of the included articles is possible. Furthermore, as mentioned above, available studies addressing the issue of drug-resistant nosocomial infections in COVID-19 settings were mainly retrospective and monocentric, often missing a control group and providing anecdotical information. Yet, the COVID-19 pandemic led to such a profound change of healthcare practices that will surely affect the patterns of antimicrobial resistance worldwide, and the present work offers a broad overview on this topic.

## 5. Conclusions

SARS-CoV2 pandemic lead to a massive disruption of healthcare systems at all levels. In acute and intensive care settings, inappropriate antimicrobial exposure, and discontinuation of infection control measures may drive selection and diffusion of drug-resistant pathogens. In COVID-19 settings, microorganisms that pose particular risk of nosocomial outbreaks are carbapenem-resistant *A. baumannii*, methicillin-resistant *S. aureus*, carbapenem-resistant *K. pneumoniae,* and *C. auris*. 

In conclusion, based on the literature review, we identified an urgent need of high-quality, controlled, perspective studies addressing the issue of antibiotic resistance in COVID-19 settings, with special focus on COVID-19 ICUs that may be new drivers of AMR selection. Novel approaches to stewardship must adapt to COVID-19 settings and local epidemiology and, most importantly, a new focus on adherence to IPC measures is urgently needed. Antimicrobial resistance will continue to pose a substantial threat to healthcare systems for the years to come. To mitigate the potential long-term impact of COVID-19 on AMR, it is necessary to integrate antimicrobial stewardship activities in the pandemic response.

## Figures and Tables

**Table 1:** Summary of the main results.

Pathogen	Region	Pre-Pandemic Trend (2015–19) ^a^	Study Type	Impact of COVID-19, Main Findings	Main Conclusions	Reference
Methicillin-resistant *S. aureus*	EU, France	Decrease	Prospective, observational	11/25 of early bacterial co-infections were due to *S. aureus,* of which 20% were MRSA.	High proportion of *S. aureus* co-infections	Elabbadi et al.
	USA, New York	Decrease	Retrospective cohort	Prevalence of MRSA on respiratory cultures increased from 0.6 at day 3 to 5.7% at day 28 of hospitalization. MRSA PCR positive tests: 12/122 (100% negative predictive value)	The findings are keeping with decreasing rates of MRSA infections across the United States	Punjabi et al.
	Mixed	N/A	Meta-analysis	Over 18 studies, the meta-analysis found a 25.6% prevalence of COVID-19/*S. aureus* co-infections. MRSA accounted for 53.9% of those infections.	High proportion of MRSA co-infections	Adeiza et al.
	EU, Italy	Decrease	Monocenter, case-control study	Among 40 COVID and non-COVID-19 ICU patients who developed ventilator-associated pneumonia due to *S. aureus* (SA-VAP), COVID-19 patients were more likely to develop methicillin-resistant infection (26/40 (65%) vs. 22/80 (27.5%); *p* < 0.01).	COVID-19 seemed to significantly affect microbiological and clinical features of SA-VAP	De Pascale et al.
	USA, New York	Decrease	Retrospective, multicenter case-series	*S. aureus* bacteremia during COVID-19 admission was seen in 1.6% (42/2679) of patients. 19/42 (45.2%) patients who developed *S. aureus* bacteremia were infected by MRSA, and in 19% of cases it originated in the lungs.	*S. aureus* bacteremia found to be uncommon but associated with high mortality rates	Cusumano et al.
Vancomycin-resistant *Enterococcus* spp.	EU, Germany	Increasing	Retrospective, monocenter case-series	Whole genome sequencing revealed close genetic relatedness between five VRE (E. faecium) isolated from patients and 11 VRE isolated from environment in a COVID-19 ICU	Importance of IPC measures to prevent VRE transmission	Kampmeier et al.
	EU, Italy	Increasing	Retrospective, monocenter	Authors noted unprecedented high BSI incidence of (87/1000 d of ICU stay). Gram-positive bacteria accounted for 79.6% of episodes, with Enterococcus species being the most prevalent (55.8%). Thirty-two isolates (27.3%) showed multidrug resistance.	Increased rate of BSI due to *Enterococcus* spp. Role of IPC measures disruption	Bonazzetti et al.
	EU, Spain	Increasing	Monocenter, case-control study	Among 91 cases of co-infections, the most frequent bacterium among patients with primary BSI was *Enterococcus faecium* (43%). Multidrug resistance was detected in 75% of the isolates.	Increased rate of superinfections due to *Enterococcus* spp. Importance of IPC measures.	Bardi et al.
Carbapenem- resistant Enterobacteriaceae	Mixed	NA	Scoping review	The results indicate the need for attention to infections caused by carbapenem-resistant K. pneumoniae. There is also a need for the ongoing surveillance and control of hospital- acquired infections. The critically ill patients are the most susceptible to infection.	Unexpected high incidence of *C. auris* infections. Importance of environmental contamination.	Medrzycka-Dabrowska et al.
	China	Unknown	Retrospective, monocenter	Higher rate of CRE colonization in patients undergoing positioning, if compared to patients who were not prone-positioned (67% vs. 37%)	High incidence and mortality of CRE secondary infections.	Yang et al.
	Peru	Unknown	Retrospective, monocenter	We reported an outbreak of Klebsiella pneumoniae New Delhi metallo-β-lactamase (NDM) in a Peruvian hospital where no cases of strains with this resistance had been identified previously	COVID-19 patients have a higher risk of acquiring MDR bacterial infections.	Arteaga-Livias et al.
	USA, New York	Stable	Retrospective, monocenter	Outbreak of five cases of nosocomial infection due to NDM-beta- lactamase producing Enterobacterales among COVID-19 patients	High incidence of CRE superinfection. Importante of AMS and IPC programs.	Nori et al.
Carbapenem resistant *A. baumannii*	India	Unknown	Retrospective, multicenter	Out of 17,534 patients admitted among 10 indian hospitals, 3.6% of patients developed secondary bacterial or fungal infections. Gram-negative bacteria were isolated from 78% of patients. *K. pneumoniae* (29%) was the predominant pathogen, followed by *A. baumannii* (21%). carbapenem resistance rates were, respectively, 92.6% and 72.8%.	High incidence of superinfection due to G- MDR pathogens. Importance of AMS and IPC programs.	Vijay et al.
	Israel	Unknown	Retrospective, monocenter	Outbreak of five infections due to CRAB on both COVID-19 ICU and general ward. The suspected mechanism of transfer was an environmental source that was transmitted through healthcare workers’ hands or equipment.	Increased rate of CRAB secondary infections. Importance of AMS and IPC programs.	Gottesman et al.
	EU, Italy	Increasing	Monocenter, case-control	In ICU, *A. baumannii* colonization was higher in COVID-19 vs. non-COVID-19 patients (63% vs. 8%), and was independently associated with a higher risk of MDR infection (RR 7.9) and 30-day mortality (OR 7.1).	Increased rate of CRAB secondary infections. Importance of AMS and IPC programs.	Russo et al.
	EU, Spain	Increasing	Retrospective, monocenter	Among all patients admitted to COVID-ICU between 1 March—31 May 2020, 16.5% of all secondary infections (22/134) were due to drug-resistant *A. baumannii*.	Increased rate of CRAB secondary infections.	Nebreda-Mayoral et al.
	Brasil	Unknown	Retrospective, monocenter	Outbreak of 14 infections due to CRAB in a new COVID-19 ICU between September and December 2020.	Increased rate of CRAB secondary infections. Role of IPC disruption.	Shinohara et al.
	Iran	Unknown	Prospective, monocenter	Among 19 patients admitted to COVID ICU, *A. baumannii* was responsible of 90% of secondary infections. Mortality was 95%.	Increased rate of CRAB and MRSA secondary infections.	Sharifipour et al.
Carbapenem-resistant *P. aeruginosa*	EU, Italy	Decreasing	Prospective, monocenter	One-hundred and eighteen patients admitted to COVID-19 ICUs were included in the study. Among them, 12 (10.2%) became colonized/infected with CRPA, 6 (5.1%) with *C. auris,* and 2 (1.6%) with CR-Kp.	Increased rate of secondary infections due to G- MDR pathogens and *C. auris*.	Magnasco et al.
Triazole-resistant *Aspergilluss* spp.	Eu, France, The Netherlands, Ireland	Unknown	Case reports	Cases presented by Meijer et al. and Mohamed et al. harbored the TR34/L98H mutation. All patients presented in these reports died.	Importance of inappropiate empirical therapy on mortality.	Ghelfenstein-Ferreira et al. Meijer et al. Mohamed et al.
*Candida auris*	EU, Italy	Unknown	Monocenter case series	Nosocomial cluster of 10 *C. auris* infections. Phylogenetic molecular clock analyses predicted a recent introduction (May 2019) in the hospital setting and suggested that most transmissions were associated with a ward converted to a COVID-19-dedicated ICU during the pandemic.	Spread of *C. auris* in Italy could have been facilitated by the COVID-19 pandemic.	Di Pilato et al.
	Brazil	Unknown	Prevalence investigation	Among body swabs collected from 47 patients, eight (n = 8/47, 17%) samples from the axillae were positive for *C. auris.* In multivariate analysis, having a colonized digital thermometer was the only independent risk factor associated with *C. auris* colonization.	Increased incidence of *C. auris* superinfection. Importance of environmental contamination.	Nobrega de Almeida Jr et al.
	India, New Delhi	Unknown	Retrospective, monocenter	Candidemia affected 15 critically ill coronavirus disease patients admitted to an intensive care unit during April–July 2020. *C. auris* accounted for two thirds of cases. Case-fatality rate was 60%.	Increased incidence of *C. auris* superinfection.	Chowdhary et al.
	Mexico	Unknown	Retrospective, monocenter	Outbreak of *C. auris* infections that started in a non-COVID patient and then spread to 12 patients in a COVID-19 ICU. Mortality was 66% among all patients and 83% among patients who developed candidemia.	Increased incidence of *C. auris* superinfection. Importance of AMS and IPC measures.	Villanueva-Lozano et al.

^a^ pre-pandemic trend, when available, is the one reported by the European Center of Disease prevention and Control [6], the World Health Organization [8], and the Center for Disease prevention and Control [7]. MRSA: methicillin-resistant S. aureus; ICU: intensive care unit; VRE: vancomycin-resistant *Enterococcus* spp.; IPC: infection prevention and control; BSI: bloodstream infection; NDM: New-Delhi Metallo beta-lactamases; AMS: antimicrobial stewardship; MDR: multidrug-resistant; CRAB: carbapenem-resistant *A. baumannii*.

## Data Availability

All data will be made available upon reasonable request.
